# Acral Melanocytic Neoplasms: A Comprehensive Review of Acral Nevus and Acral Melanoma in Asian Perspective

**DOI:** 10.3390/dermatopathology9030035

**Published:** 2022-08-19

**Authors:** Sanghyun Park, Sook-Jung Yun

**Affiliations:** 1 Department of Dermatology, Chonnam National University Hwasun Hospital, Hwasun 58128, Korea; 2 Department of Dermatology, Chonnam National University Medical School, Gwangju 59626, Korea

**Keywords:** acral melanocytic nevus, acral melanoma, clinicopathological feature, genetic alteration, treatment

## Abstract

Acral melanocytic neoplasms, including acral melanocytic nevus and acral melanoma, are common melanocytic lesions in Asian populations. Both lesions occur on the volar surface of the hands and feet, and on nail units. Acral melanocytic nevi occur on the arch area of the sole, whereas acral melanomas frequently occur on weight-bearing areas of the sole, and on the fingernails. Therefore, the development of acral melanoma may be associated with chronic pressure, physical stress, or trauma. Dermoscopy is a useful adjunctive diagnostic tool for differential diagnosis. Acral melanocytic nevus is characterized by a parallel furrow pattern, whereas acral melanoma has a parallel ridge pattern. Genetic alterations are also different between the two types of lesion. *BRAF* and *NRAS* mutations are common in acral melanocytic nevus, whereas acral melanoma shows lower rates of *KIT*, *NF1*, *BRAF*, and *NRAS* mutations and remarkable copy number variations in genes such as *CCND1*, *CDK4*, *hTERT*, *PAK1*, and *GAB2*. Sentinel lymph node biopsy is important for staging and prognosis. Contemporary treatments for melanoma include targeted therapy for mutations and immunotherapy, such as anti-PD1 inhibitors.

Melanocytic neoplasms in Asian populations are characterized by a predominance of acral melanoma compared with populations from Western countries. Here, we focus on acral melanoma and its benign counterpart, acral melanocytic nevus.

## 1. Acral Melanocytic Nevus

Acral melanocytic nevus occurs on the palms of the hands, soles of the feet, and nail units.

### 1.1. Epidemiology

A study performed in the USA in 2010 indicated prevalence rates of acral melanocytic nevus of 23.0% and 42.0%, in White and Black populations, respectively [1]. A more recent large prospective cohort study (performed in 2016) analyzed a much larger number of cases, including Asian and Hispanic populations, and showed that 36% of people in the United States have at least one acral nevus [2]. Interestingly, the total number of melanocytic nevus and their distribution at the anatomic sites are different according to racial populations. A Brazilian study reported that patients with darker skin have fewer overall acquired nevi, but more nevi on the face and acral sites, than patients with lighter skin [3]. In Japan, acral melanocytic nevi was reported in approximately 7% in the population, and 10.9% of subjects in another study had melanocytic nevi on the soles of the feet [4,5]. The rates of acral nevus on the palms and soles in a Korean population were reported to be 15.7% and 9.2%, respectively [6]. In summary, acral melanocytic nevi occur more commonly in Asians and people of darker skin types compared with lighter skinned individuals, even though the overall prevalence of melanocytic nevi is lower in the former than the latter. The limitation of these data is they analyzed only patients who visited dermatologic clinic; therefore, the possibility of selection bias may exist.

### 1.2. Etiology and Genetics

The etiology of acral melanocytic nevus is still unknown. Numerous genetic studies have been conducted on melanocytic nevi in low-cumulative sun-damaged (low-CSD) skin, but the genetic profiles of those in acral sites are still unclear. Genomic analyses have shown that most nevi on low-CSD skin harbor mutually exclusively mutations in melanoma driver oncogenes, such as *BRAF* and *NRAS* [7,8,9]. One author (S.J.Y.) and coworkers also found mutations in five genes in acral melanocytic nevi in Korean subjects, i.e., *BRAF* (66.7%), *NRAS* (9.5%), *NF1* (9.5%), *GNAQ* (38.1%), and *KIT* (14.3%) [10]. *BRAFV600E* and *NRAS* mutations were mutually exclusive, whereas *GNAQ* mutation co-occurred with other gene mutations. Copy number variations (CNVs) are more common in acral melanoma than non-acral cutaneous melanoma [11]. They showed that CNVs were much less common in acral melanocytic nevus than acral melanoma. A mutational survey of acral melanocytic nevi was also performed in a U.S. population [12]. Similarly to the Korean results, *BRAF* mutation was commonly observed in acral melanocytic nevus. In that study, mutations in *BRAF* and *NRAS* were observed in 86% and 10% of acral melanocytic nevi, respectively. These mutational profiles were similar to benign melanocytic nevi in low-CSD skin. Mutations in *BRAF* and *NRAS* were mutually exclusive. Very low levels of CNV were observed in acral melanocytic nevus compared with acral melanoma. Therefore, it was concluded that acral melanocytic nevi demonstrated a mutational spectrum similar to nevi on low-CSD skin, suggesting that they are unlikely to be precursor lesions for the majority of acral melanomas. Further large-scale studies are needed to draw definitive conclusions regarding this issue.

### 1.3. Clinical Features

**Figure 1:**
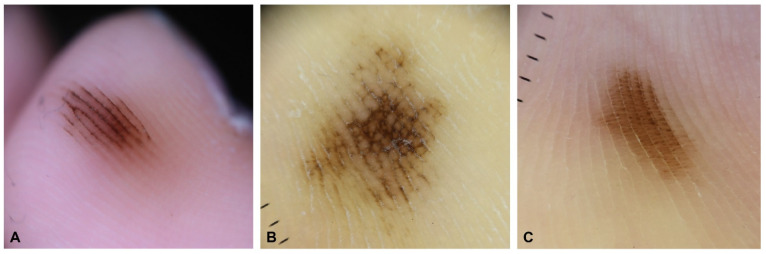
Three major dermoscopic patterns in acral melanocytic nevus. (**A**) Parallel furrow pattern. (**B**) Lattice-like pattern. (**C**) Fibrillar pattern.

Acral melanocytic nevus occurs on the palms of the hands, soles of the feet, and nails, all of which are non-hair-bearing sites. These nevi may also occur on the volar surface of the fingers and toes. Acral melanocytic nevi have a different distribution on volar sites than acral melanomas, and primarily affect non-weight-bearing areas [13,14,15]. Most of these lesions present as brown-to-black small (less than 6 mm) macules or papules. They are typically symmetrical and well circumscribed, although they are sometimes asymmetrical and poorly circumscribed. These atypical acral nevi are difficult to diagnose, both clinically and pathologically, because early acral melanoma may have a similar clinical presentation. In such cases, dermoscopy is a useful noninvasive tool for the differential diagnosis of acral pigmented lesions [16,17,18]. Acral melanocytic nevi have three major dermoscopic patterns: a parallel furrow pattern, lattice-like pattern, or fibrillar pattern (Figure 1). The parallel furrow pattern is regarded as the prototype of the three major dermoscopic patterns of acral melanocytic nevus. Melanocytes (nevus cells) are mostly arranged in well-demarcated nests mainly located in the crista profunda limitans underlying the surface furrow [19]. These melanocytic nests form melanin columns in the cornified layer under the sulci of the surface skin. This histopathological finding mirrors the parallel furrow pattern shown by dermoscopy. The fibrillar pattern is caused by an oblique arrangement of melanin pigment in the slanting cornified layer, which is induced by mechanical pressure from the body weight [20]. Therefore, the fibrillar pattern can be regarded as an artifactual expression of the parallel furrow pattern [16].

**Figure 2:**
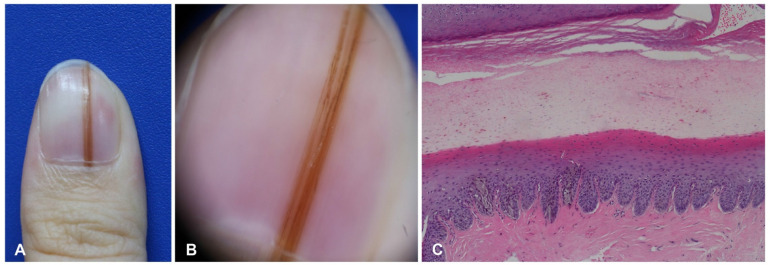
Nail matrix nevus in a 28-year-old woman. (**A**) Brownish linear melanonychia of the nail plate. (**B**) Regular line on dermoscopy. (**C**) Junctional nests in the epidermis of the nail matrix.

**Figure 3:**
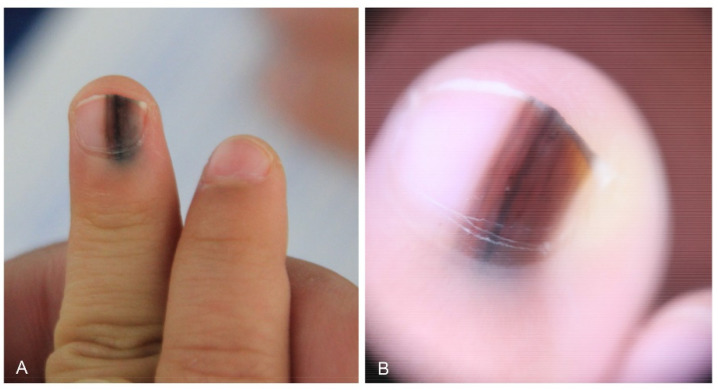
Nail matrix nevus in a 2-year-old girl. (**A**) Wide melanonychia on the 3rd fingernail. (**B**) Dermoscopy showing irregular multicolored lines.

Nail matrix nevus, which is an acral melanocytic nevus located on the nail matrix, manifests as longitudinal melanonychia. This pigmentation of the nail plate is mostly derived from nevus cells on the distal nail matrix (Figure 2). Dermoscopy can be useful for visualizing these findings. This longitudinal band exhibits regularly spaced, thickened, parallel lines on a brownish background, running from the proximal to distal direction on the nail plate. However, nail matrix nevus in children manifests with atypical features, including irregular wide, dark, and multicolored lines (Figure 3) and micro-Hutchinson’s sign [21,22]. The micro-Hutchinson’s sign is defined by the visibility on dermoscopy of a pigmentation of the periungual tissues that could not be seen with the naked eye [23].

### 1.4. Histopathological Features

**Figure 4:**
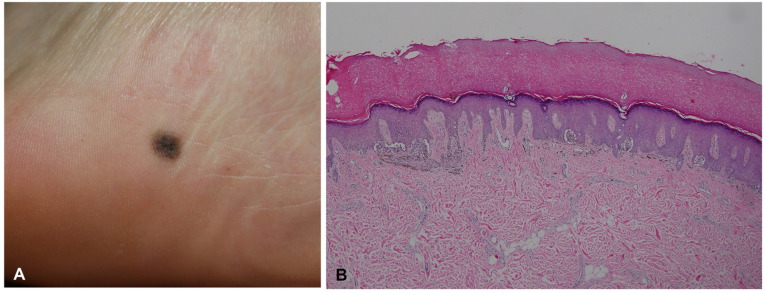
Acral melanocytic nevus in a 23-year-old woman. (**A**) Small round macule on the inner heel. (**B**) Histopathological analysis revealed bridging formation of rete ridges with junctional melanocytic nests.

Acral melanocytic nevus presents as a junctional or compound melanocytic nevus, characterized by relatively small, symmetric, and well-circumscribed lentiginous and nested melanocytic proliferation along the dermal–epidermal junction (Figure 4). Nevus nests are variable in size and often vertically oriented. Pagetoid scatter, bridging between rete ridges, and fibroplasia are not uncommon, but these features tend to be confined to the center of the lesion [13,24]. When dermal components are present, maturation with depth and lack of mitotic activity on deeper portions are usually observed. Nevus cells occasionally show mild cytological atypia.

Nail matrix nevus is usually junctional, and compound nevus is rare [25,26,27]. Nuclear atypia, confluence of melanocytes, focal pagetoid spread, and periungual involvement are common in children, which can lead to difficulties in the interpretation of histological specimens [24,28].

### 1.5. Management of Acral Melanocytic Nevus

The lesions are benign, and progression to melanoma is rare. Most acral melanomas arise de novo, not in association with a preexisting acral nevus [12,29]. Acral melanocytic nevi which do not show typical benign dermoscopic patterns may require clinical and dermoscopic surveillance, and once or twice a year is enough [30]. Biopsies can be considered if the lesion enlarges to more than 7 mm, or changes in size and shape on regular follow-up. Pediatric patients with longitudinal melanonychia can be followed up without intervention for several years even if lesions grow darker or wider, whereas only 5% completely regress within 4.5 years [31]. Decisions should be based on clinicodermoscopic and pathologic correlations.

## 2. Acral Melanoma

Acral melanoma is a subtype of cutaneous melanoma occurring in glabrous acral skin, such as the palms, soles, and nail units, especially the nail matrix [32]. The most common clinicohistological type is acral lentiginous melanoma based on the lentiginous pattern on histology, and was first discussed by Reed in 1976. Subungual melanoma, which was first described as melanotic whitlow by Hutchinson in 1886 [33], is an acral melanoma occurring in the nail matrix. The recent WHO Classification of Skin Tumours presents nine pathways of melanoma; acral melanoma is pathway V [34].

### 2.1. Epidemiology

Acral melanoma is rare in Caucasians, who have a high overall incidence of melanoma. In the USA, acral melanoma accounts for only 2–3% of all malignant melanomas [35], whereas it accounts for a high proportion of melanomas in Asian and African populations. In Korea, approximately 50% of melanoma patients have acral melanoma [36,37,38,39] which is similar to the proportions in other Asian populations, including Japan [40], Singapore [41], China [42], and Taiwan [43]. Although the relative proportion of acral melanoma varies, its absolute incidence is similar in all races [44]. Subungual melanomas affect the fingernails more frequently than the toenails, and the fingernails of the right hand are more susceptible than those of the left hand. The thumbnail and great toenail are more common sites than other digits [34]. We speculate that trauma or external factors might be associated with this location preference.

### 2.2. Etiology and Genetics

The precise etiology of acral melanoma has not been determined. In a study in White populations, acral melanoma was strongly associated with high total body nevus count and nevi on the soles of the feet [45]. However, in a Japanese study, although a high number of acquired nevi was a risk factor for nonacral melanoma development, acquired nevi on the soles, palms, and nail apparatus did not seem to be a risk factor for the development of acral melanoma [46]. In the abovementioned study of a White population, there were also significant associations with penetrative injury of the feet or hands and heavy exposure to agricultural chemicals [45]. Several recent studies, including ones conducted in our department, revealed an association of acral melanoma on the plantar or palmar surfaces with mechanical or physical stress through anatomical mapping [15,47,48]. Analysis in 313 Korean acral melanoma patients revealed that 26.8% reported traumatic events, and 29.1% experienced physical stress. The most common occupations were farmers and fishermen [49]. Physical stress, pressure, friction, maceration, irritation, and trauma may play roles in the pathogenesis of acral melanoma, and further studies are required to precisely determine the underlying molecular mechanism.

Genetic analysis showed that acral melanoma is characterized by a relatively low mutation burden and a relatively high frequency of CNVs [11,50,51]. In addition to oncogenic mutations of *BRAF* (10–35%) and *NRAS* (9–22%), which are mutually exclusive, as well as *NF1* (11–23%), triple-wild-type driver mutations (45–58%) were observed [50] Bastian et al. [52] reported that genetic aberrations of *KIT* frequently occur in acral melanoma. In Koreans, *KIT* mutations and increased *KIT* copy numbers are commonly observed in acral melanoma, and *KIT* mutations were found to be independent risk factors for a poor prognosis [38]. Identified CNVs in acral melanoma have implicated several genes, including *CCND1*, *CDK4*, *hTERT*, *PAK1*, *GAB2*, *EP300*, *YAP1*, and *MDM2* [32,50,51,53]. Deletions in *PTEN* and *CDKN2A* are seen in up to 25% of cases [51]. The earliest genetic alteration in acral melanoma is the amplification of *CCND1*, which is detected in the very early stages of acral melanoma in situ [11,49]. In Taiwan, *NRAS*/*KRAS* mutations, cell cycle aberrations, increased copy numbers in antiapoptotic genes *BIRC2*, *BIRC3*, and *BIRC5*, and the amplification of receptor tyrosine kinase genes were significantly enriched in acral melanoma [54]. Further genetic profiling is needed to understand the pathogenesis of acral melanoma and identify suitable agents for targeted therapy.

### 2.3. Clinical Features

**Figure 5:**
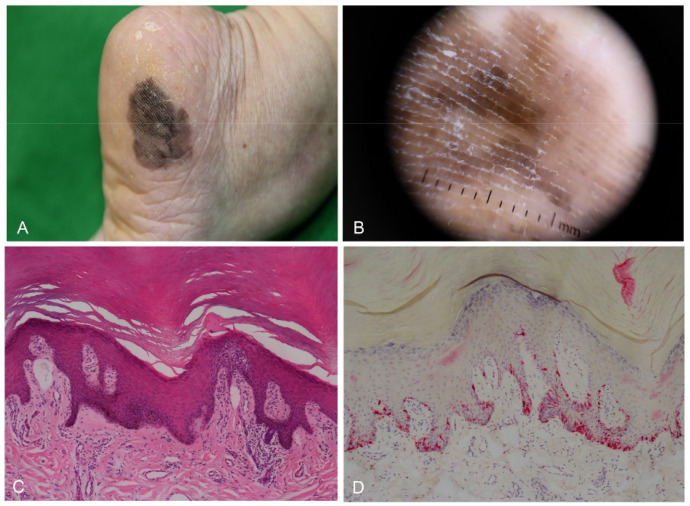
Acral melanoma in situ in a 68-year-old woman. (**A**) Large irregular black patch on the outer area of the heel. (**B**) Dermoscopy showing parallel ridge pattern. (**C**) Skin biopsy showing a slight increase in basal pigmentation with mild lymphocytic infiltrate (H&E staining, original magnification, ×100). (**D**) Melan-A immunostaining highlights lentiginous proliferation of atypical melanocytes with prominent dendrites (×100).

Acral melanoma begins as an asymmetrical, black-to-brown macule or small irregular patch. At the time of biopsy, the lesion is typically large in size after a long radial growth phase (RGP), and elevated nodules and ulceration associated with the vertical growth phase (VGP) may occur [34]. Acral melanoma on the sole primarily occurs on the weight-bearing portions, including the heel and forefoot areas [47,48]. One study showed that acral melanoma on the palms tends to occur in the finger pulp and distal area of the distal transverse crease of the palm, and suggested that mechanical stress may also affect the occurrence of palmar melanoma [15]. Due to their long RGP and clinical similarity with acral melanocytic nevus, differential diagnoses can sometimes be difficult based only on clinical findings. Dermoscopy can increase the sensitivity and specificity of diagnosis of early acral melanoma [19]. The parallel ridge pattern, consisting of brown-to-black band-like pigmentation on the ridges of the skin on dermoscopy, was correlated with histopathological findings (Figure 5). In an early evolving acral melanoma lesion, solitary arranged melanocytes preferentially proliferated in the crista profunda intermedia, which is an epidermal rete ridge underlying the surface ridge. This can be seen in tissue sections cut perpendicular to the surface skin markings [19]. Acral melanoma sometimes manifests as nodular melanoma, i.e., as a large nodule without a surrounding pigmented patch. It may be due to the very short RGP, but the precise mechanism remains unclear. Rarely, amelanotic acral melanomas, which show little or no brown-to-black pigmentation, can occur and can easily be misdiagnosed as benign conditions [55].

**Figure 6:**
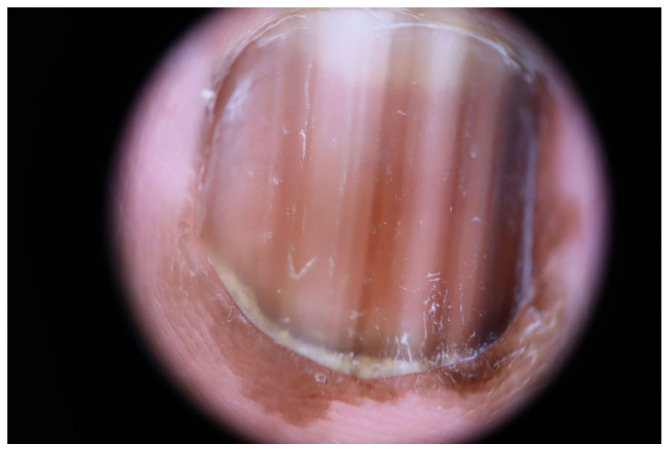
Subungual melanoma in situ in a 63-year-old woman. Dermoscopy showing irregular, multicolored lines, and Hutchinson’s sign.

Subungual melanoma often starts as longitudinal melanonychia, and a pigmented patch then spreads over the entire nail plate and into the skin beyond the nailfolds and hyponychium, which is called Hutchinson’s sign [56]. Over time, nodules, ulcers, bleeding, and nail destruction can occur. Dermoscopy is also useful for differential diagnoses of nail matrix nevus and subungual melanoma [57]. The dermoscopic features mostly indicative of early subungual melanoma are multiple irregular longitudinal brown-to-black lines on a brown background, micro-Hutchinson’s sign, a wide pigmented band, and triangular pigmentation on the nail plate (Figure 6). The sensitivity and specificity of micro-Hutchinson’s sign on dermoscopy in subungual melanoma in situ were reported to be 0.42 and 0.96, respectively [58].

### 2.4. Histopathological Features

**Figure 7:**
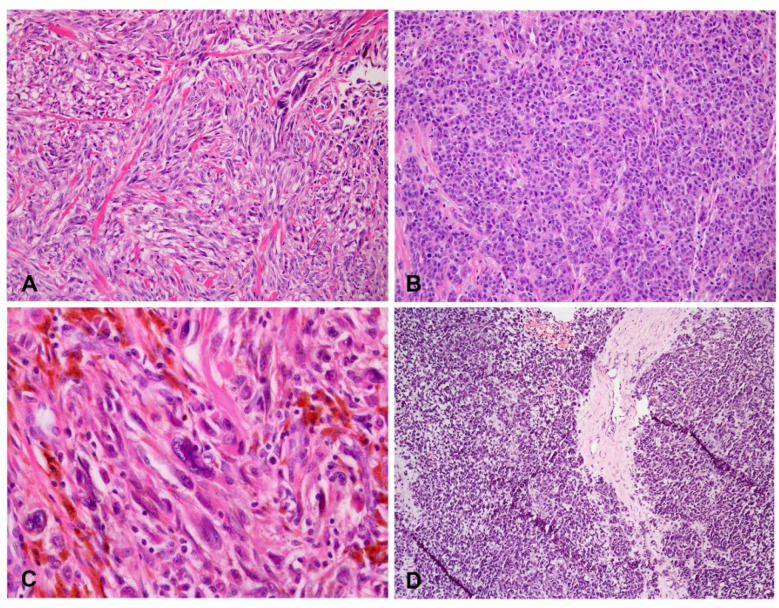
Variable dominant cell morphology in advanced acral melanoma. (**A**) Spindle cells (H&E staining, original magnification, ×200). (**B**) Epithelioid cells (H&E staining, original magnification, ×200). (**C**) Bizarre giant cells (H&E staining, original magnification, ×400). (**D**) Lymphoid cells (H&E staining, original magnification, ×100).

The most common histopathological subtype of acral melanoma is acral lentiginous melanoma, but nodular melanoma and superficial spreading melanoma can be seen in acral sites. The histopathological and molecular features of superficial spreading melanoma and nodular melanoma on acral sites are similar to those of examples at other body sites. Superficial spreading melanoma occurs mostly in the dorsal surface of acral sites, but rarely on volar surfaces [59]. Nodular melanoma on acral sites represent VGP of acral melanoma without an RGP. In the very early stages of acral lentiginous melanoma, there are only scattered lentiginous atypical melanocytes with enlarged hyperchromatic nuclei and prominent dendrites in the epidermal basal layer [60]. Over time, the continuous lentiginous proliferation of atypical melanocytes is observed, and after a long period of RGP, VGP occurs and the dermal proliferation of atypical melanocytes with large numbers of mitotic figures may be seen. The dominant cell morphology is variable in advanced acral melanoma (Figure 7). One author (S.J.Y.) and coworkers found correlations of the cytomorphological features and mutation status of acral melanomas. Round epithelioid cells are common in cases with *BRAF* mutation, bizarre giant cells are associated with *NRAS* mutation, and spindle cells with prominent dendrites are frequently observed in cases with *NF1* and *GNAQ* mutations [10]. Histopathological features may be desmoplastic, neurotropic, or syringotropic. Immunohistochemical staining for HMB45 and Melan-A highlights prominent dendritic processes of melanoma cells in the epidermis of acral lentiginous melanoma, whereas HMB45 is sometimes focally positive or negative in amelanotic melanoma [55]. Recent studies have evaluated the role of preferentially expressed antigen in melanoma (PRAME) and p16 immunohistochemical staining in acral melanocytic neoplasm [61]. There are strong expressions of PRAME in acral melanomas, compared with weak or negative expression in acral melanocytic nevi. PRAME expressions are strong in both subungual and non-subungual acral melanomas [62]. Loss of p16 is also helpful to the diagnosis of acral melanoma.

### 2.5. Staging and Treatment

Similar to other cutaneous melanomas, acral melanoma is staged according to the American Joint Committee on Cancer (AJCC) TNM (tumor, node, metastasis) criteria [63]. However, compared with similar T stages of non-acral melanomas, acral melanomas have higher locoregional recurrence rates and poorer survival rates [64]. Previously, the poor outcomes of acral melanoma were explained by delays in diagnoses, but several recent studies have shown that acral melanoma has a high rate of locoregional metastasis and poorer survival outcomes than nonacral melanoma, even after controlling for melanoma stage.

The National Comprehensive Cancer Network recommends sentinel lymph node biopsy (SLNB) for primary melanomas with AJCC stage T1b or greater [65]. SLNB may be particularly important for acral melanoma because of its high locoregional recurrence rate. Patients with a positive SLNB are at higher risk of recurrence and should be followed via nodal basin ultrasound surveillance without complete lymph node dissection, given the lack of any improvement in melanoma-specific survival in two randomized controlled trials comparing the merits of complete lymph node dissection following a positive SLNB versus clinical observation with nodal ultrasound [66]. Recently, one author (S.J.Y.) and coworkers reported that the degree of acral melanoma pigmentation was a key predictor of metastasis. Amelanotic acral melanomas, and those with mild pigmentation, were associated with a first lung metastasis, whereas heavy pigmentation was associated with first lymph node metastasis [67,68]. These observations may be relevant to the initial workup, i.e., in cases of melanoma with low pigmentation, full-body imaging examination may be necessary at an early stage.

Wide radical excision is the standard treatment for primary acral melanoma, similar to other cutaneous melanomas. Current recommendations regarding the clinical margins differ according to the Breslow thickness of the primary lesion, and are based on several large randomized trials comparing margins of different size: margins of 0.5–1 cm are recommended for melanoma in situ, whereas 1 cm is recommended for melanoma with Breslow depth <  1 mm, 1–2 cm for melanoma 1–2 mm thick, and 2 cm for melanoma ≥ 2 mm thick [69]. However, acral melanoma was not included in the randomized trials on which the guidelines are based; thus, questions remain regarding whether the current guidelines should continue to be applied to acral melanoma. In addition, the management of acral lentiginous melanoma presents surgical challenges because of the subclinical extension of tumor cells and anatomically constrained locations. Therefore, Mohs micrographic surgery with complete circumferential peripheral and deep margin assessment has been examined as a means to improve histological clearance and reduce the chance of local recurrence in comparison with conventional wide excision [66].

*BRAF* mutation is an important driver of mutation in melanoma; therefore, *BRAF* inhibitors have been used to treat metastatic melanoma and recurrent melanoma with *BRAF* mutation. However, because *BRAF* mutation is less common in acral melanoma, *BRAF* inhibitors are not generally useful for their treatment. Positive responses to *KIT* inhibitor treatment have been reported in cases of acral melanoma with *KIT* mutation [70]. The introduction of immune checkpoint inhibitors has changed the management of metastatic melanoma. However, lower response rates to immune checkpoint blockades have been observed in acral melanoma and nail unit melanoma, which may be due to smaller numbers of tumor-infiltrating lymphocytes, lower PD-L1 expression, and a lower mutational burden in acral melanoma compared with other subtypes of melanoma [71].

## 3. Conclusions

Acral melanocytic nevus and acral melanoma are frequent melanocytic neoplasms in Asian populations; therefore, precise diagnoses and a detailed understandings of treatment options are essential.
